# Real-world comparative effectiveness of faricimab versus aflibercept 2 mg in treatment-naïve exudative neovascular AMD patients treated with a treat-and-extend regimen

**DOI:** 10.1038/s41433-026-04710-y

**Published:** 2026-08-04

**Authors:** Xuenan Zhuang, Lorena Ulla, Claudio Foti, Rossella Cariola, Giovanni Neri, Chiara Olivieri, Guglielmo Parisi, Francesco Petrillo, Paola Marolo, Michele Reibaldi, Enrico Borrelli

**Affiliations:** 1 Guangdong Cardiovascular Institute, Guangdong Provincial People’s Hospital Ganzhou Hospital, Guangdong Academy of Medical Sciences, Guangzhou, China; 2 Guangdong Eye Institute, Guangdong Cardiovascular Institute, Department of Ophthalmology, Guangdong Provincial People’s Hospital (Guangdong Academy of Medical Sciences), Southern Medical University, Guangzhou, China; 3 Department of Surgical Sciences, University of Turin, Turin, Italy; 4 Department of Ophthalmology, “City of Health and Science” Hospital, Turin, Italy; 5 Department of Ophthalmology, Faculty of Medicine, Biruni University, İstanbul, Turkey

**Keywords:** Prognostic markers, Retinal diseases

## Abstract

**Purpose:**

To compare the real-world effectiveness of faricimab versus aflibercept 2 mg in treatment-naïve patients with neovascular age-related macular degeneration (nAMD).

**Methods:**

We analysed treatment-naïve nAMD patients aged ≥50 years initiating intravitreal anti-VEGF therapy between March 2024 and September 2024. Patients received either faricimab or aflibercept 2 mg. Inverse probability of treatment weighting was used to minimise selection bias. Primary outcomes were best-corrected visual acuity (BCVA) changes from baseline to post-loading phase and 1-year follow-up. Secondary outcomes included anatomic resolution of intraretinal fluid (IRF), subretinal fluid (SRF), and subretinal hyperreflective material (SHRM), treatment burden, and safety.

**Results:**

A total of 172 patients were included (86 faricimab, 86 aflibercept). Baseline characteristics were well-balanced between groups. The faricimab regimen was associated with greater BCVA improvement compared with aflibercept at post-loading phase (adjusted mean difference -0.075 logMAR; 95% confidence interval [CI], -0.130 to -0.020; *P* = 0.008) and at 1 year (-0.073 logMAR; 95% CI, -0.128 to -0.018; *P* = 0.011). Patients receiving faricimab achieved significantly higher rates of SRF resolution (odds ratio 2.446; 95% CI, 1.012 to 5.912; *P* = 0.047). The faricimab group was associated with fewer injections from the post-loading phase to 1 year (median 3 vs. 4; *P* < 0.001) and lower therapy switching rates (7.9% vs. 25.6%; *P* = 0.003).

**Conclusions:**

In treatment-naïve nAMD patients, faricimab achieved greater visual acuity gains, enhanced anatomic outcomes, and reduced treatment burden compared with aflibercept. However, these findings should be interpreted in light of the non-randomised design, different loading regimens, and potential differences in early treatment exposure.

## Introduction

Neovascular age-related macular degeneration (nAMD) represents a leading cause of irreversible severe vision loss globally [1]. The advent of intravitreal anti-vascular endothelial growth factor (anti-VEGF) therapy has revolutionised the management of this condition, fundamentally altering the treatment paradigm [2, 3]. Over the past decade, anti-VEGF agents have become the established first-line standard of care for nAMD, demonstrating significant efficacy in clinical practice [4–7]. Among these, aflibercept 2 mg has emerged as a mature and extensively utilised therapeutic, with its robust efficacy and safety profile supported by a wealth of clinical evidence, making it a common benchmark in both clinical trials and routine care [5, 8, 9]. A higher dose of aflibercept (8 mg) has recently been introduced into routine clinical practice, as it has shown prolonged efficacy and a more rapid resolution of fluid in patients with nAMD [10, 11].

Faricimab introduced a novel therapeutic approach, being the first bispecific antibody for intraocular use, specifically engineered to simultaneously inhibit two distinct pathogenic pathways: angiopoietin-2 (Ang-2) and vascular endothelial growth factor A (VEGF-A) [5, 6, 12, 13]. This dual mechanism of action is theorised to confer enhanced vascular stability, potentially leading to greater potency and extended durability of treatment effect compared to agents targeting only the VEGF pathway [5, 12–14]. The pivotal phase III trials, TENAYA and LUCERNE, provided foundational evidence for its clinical utility, demonstrating that faricimab achieved visual acuity gains that were non-inferior to aflibercept 2 mg while offering the potential for extended dosing intervals of up to every 16 weeks, thereby promising a reduction in treatment burden for patients [5, 6, 9, 12, 13, 15, 16].

A well-recognised disparity exists between the controlled environment of randomised controlled trials (RCTs) and the complexities of routine clinical practice. RCTs typically employ stringent inclusion and exclusion criteria and fixed treatment protocols, which may not fully reflect the heterogeneous patient populations and flexible treat-and-extend (T&E) regimens encountered in real-world evidence (RWE) settings [17]. While a growing body of RWE for faricimab is emerging, these studies have predominantly focused on refractory cases or patients being switched from other anti-VEGF therapies due to a suboptimal response or high treatment burden, rather than on treatment-naïve populations [18–24]. Consequently, there is a clear knowledge gap regarding the comparative effectiveness of faricimab and aflibercept 2 mg as first-line therapies in a real-world context for treatment-naïve nAMD.

Therefore, the objective of this study was to address this gap by directly comparing the outcomes of faricimab and aflibercept 2 mg as first-line treatments within a real-world cohort of treatment-naïve patients with nAMD. Patients in both groups received treatment following a T&E regimen. This investigation focuses on comparing key endpoints, including visual function outcomes, anatomical responses as assessed by optical coherence tomography (OCT), and overall treatment burden.

## Methods

### Study design and setting

This was a single-centre, retrospective, comparative cohort study conducted at the Department of Ophthalmology, “City of Health and Science” Hospital, Turin, Italy. The study was performed in accordance with the tenets of the Declaration of Helsinki. Due to the retrospective nature of the analysis of de-identified data, the local institutional review board approved the study and granted a waiver of the requirement for patient informed consent. The reporting of this study adheres to the Strengthening the Reporting of Observational Studies in Epidemiology (STROBE) guidelines.

### Patient population and eligibility criteria

We identified all treatment-naïve patients with nAMD who initiated intravitreal anti-VEGF therapy at our institution. Both cohorts included patients who started treatment between March 2024 and September 2024. Inclusion criteria were: (1) a diagnosis of treatment-naïve nAMD; (2) age 50 years or older; and (3) completion of the loading phase and initiation of a T&E regimen. Exclusion criteria were: (1) any prior treatment for nAMD, including other anti-VEGF agents or photodynamic therapy; (2) the presence of other ocular pathologies that could affect visual acuity, such as pachychoroid disease spectrum, diabetic retinopathy, retinal vein occlusion, glaucoma, or significant cataract; (3) patients unable to cooperate with visual acuity testing; or (4) significant media opacities that precluded high-quality OCT imaging.

The diagnosis of treatment-naïve exudative nAMD was established by the treating physician (E.B.) using multimodal imaging, as previously described [25–28], including spectral-domain OCT (SD-OCT) with the Heidelberg Spectralis HRA + OCT (Heidelberg Engineering, Heidelberg, Germany), swept-source OCT angiography (Plex Elite, Zeiss, Dublin, CA, USA), and, in selected cases, dye angiography (Heidelberg Engineering, Heidelberg, Germany). Dye angiography, including fluorescein angiography and indocyanine green angiography, was performed at the discretion of the treating physician (E.B.) when structural OCT and OCTA alone could not confidently establish the diagnosis or MNV subtype, or when additional confirmation of lesion activity was clinically required.

At baseline, all subjects underwent a comprehensive ophthalmologic assessment, including BCVA measurement and SD-OCT volumetric scans covering a 20×20° area centred on the fovea with 49 B-scans. Post-loading assessments were performed approximately 8 weeks after completion of the drug-specific loading phase in both treatment groups. During these visits, BCVA measurement and OCT volumetric scans with tracking were acquired to ensure evaluation of the exact same retinal regions.

### Treatment Regimens

Patients in the faricimab group received four initial monthly injections of 6 mg faricimab as a loading phase, whereas patients in the aflibercept group received three initial monthly injections of 2 mg aflibercept as a loading phase, according to the licensed regimens used during the study period. After completion of the loading phase, patients were scheduled for their first post-loading assessment approximately 8 weeks after the final loading injection. Thereafter, patients were managed using a T&E regimen in which BCVA and OCT findings were reviewed at each visit. Intervals were extended when exudative activity was controlled, maintained when disease status was stable, and shortened when recurrent or persistent fluid, new haemorrhage, or visual deterioration was observed, at the discretion of the treating retinal specialist. All patients were managed by a single treating retinal specialist (E.B.), who made treatment decisions throughout the study period according to routine clinical assessment and OCT findings.

Treatment allocation was not randomised. During the study period, the choice between faricimab and aflibercept 2 mg was determined in routine clinical practice based on drug availability, reimbursement or administrative considerations, and physician-patient discussion, rather than a prespecified study protocol. There were no formal baseline visual acuity or OCT criteria mandating assignment to either treatment.

### Data collection and study variables

Anonymised data were extracted from the electronic medical records of the study site. Baseline data included patient demographics and ocular characteristics. Ocular characteristics consisted of baseline BCVA, which was assessed as part of routine clinical care using best refractive correction and recorded primarily in decimal visual acuity notation. Occasional fractional entries were converted to decimal equivalents before transformation to the logarithm of the minimum angle of resolution (LogMAR) for analysis. Ocular characteristics also included morphological features on SD-OCT, including the presence of intraretinal fluid (IRF), subretinal fluid (SRF), subretinal hyperreflective material (SHRM), and the macular neovascularisation (MNV) subtype (Type 1, 2, or 3). OCT grading was performed by two experienced graders who were masked to treatment type and final outcomes. The graders subsequently met to reach a consensus on each case. If a single consensus could not be achieved, the senior author reviewed the case with the graders to determine the final grading.

As mentioned above, follow-up data were collected at the first visit after the loading phase (post-loading assessment) and at the 1-year visit (±1 month). Study variables included BCVA and OCT features at each timepoint, as well as treatment burden metrics such as the total number of injections, the number of injections after the loading phase, and the incidence of switching to an alternative anti-VEGF therapy. Safety outcomes, including the incidence of retinal pigment epithelium (RPE) rupture, ocular inflammation, and endophthalmitis, were also recorded.

### Study outcomes

The primary outcomes were the mean or median change in BCVA from baseline to the post-loading phase assessment and from baseline to the 1-year follow-up. Secondary outcomes included the proportion of eyes achieving complete resolution of IRF, SRF, combined IRF or SRF, and SHRM at the post-loading and 1-year timepoints. Other secondary outcomes were the mean number of injections after the loading phase, the proportion of patients requiring a switch in therapy, and the incidence of safety events.

### Statistical analysis

Baseline characteristics were compared between the two treatment groups using independent t-tests or Wilcoxon rank-sum tests for continuous variables and chi-square or Fisher’s exact tests for categorical variables, as appropriate. To account for potential selection bias and confounding inherent in a non-randomised study, we used inverse probability of treatment weighting (IPTW). A propensity score for receiving faricimab or aflibercept was calculated for each patient using a logistic regression model that included baseline age, sex, BCVA, and MNV subtype as covariates. Stabilised weights were generated and truncated at the 1st and 99th percentiles to minimise the effect of extreme weights. In the IPTW-weighted cohort, treatment effects were estimated using regression models. Weighted linear regression models were used for continuous outcomes (BCVA change), and weighted logistic regression models were used for binary outcomes (anatomic resolution, drug switching, and safety events). All models were adjusted for the same set of baseline covariates used in the propensity score model. All statistical analyses were performed using R software (Version 4.4.1; R Foundation for Statistical Computing, Vienna, Austria). A two-sided *P*-value < 0.05 was considered statistically significant.

## Results

### Baseline demographics and characteristics

**Fig. 1:**
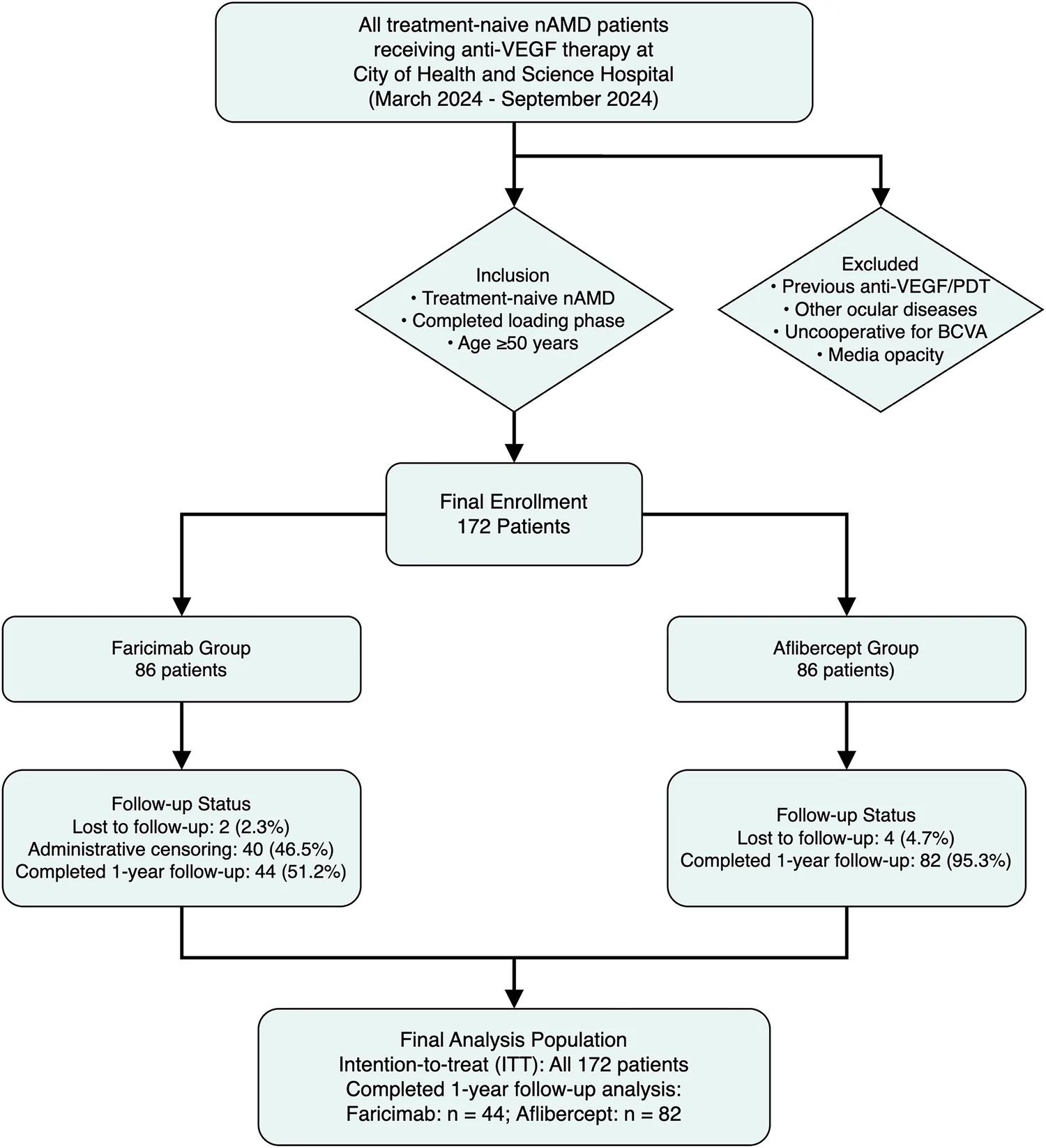
Study flowchart showing patient selection and enrolment. The flowchart illustrates the screening, enrolment, and follow-up process for treatment-naïve nAMD patients receiving anti-VEGF therapy. Inclusion and exclusion criteria are shown, along with the final treatment allocation and completion rates for both study arms. Numbers indicate patient counts at each stage of the study process. nAMD neovascular age-related macular degeneration, VEGF vascular endothelial growth factor, PDT photodynamic therapy, BCVA best-corrected visual acuity, ITT intent-to-treat.

A total of 172 treatment-naïve nAMD patients were included in this retrospective analysis, comprising 86 patients in the faricimab arm and 86 in the aflibercept 2 mg arm. Baseline demographics and ocular characteristics were well-balanced between the two treatment groups (Table 1). Dye angiography was performed in 3 of 86 eyes (3.5%) in the faricimab group and 9 of 86 eyes (10.5%) in the aflibercept 2 mg group, with no statistically significant difference between groups (*P* = 0.132). The mean (± standard deviation [SD]) age was 80.0 ± 6.4 years for the faricimab group and 79.2 ± 8.2 years for the aflibercept 2 mg group (*P* = 0.495). The proportion of male patients was 41.9% and 38.4%, respectively (*P* = 0.756). Key baseline ocular parameters, including median BCVA in LogMAR (0.400 vs. 0.430) and the distribution of MNV subtypes, were also comparable between the arms. During the follow-up period, 7 patients (8.1%) in the faricimab arm and 4 patients (4.7%) in the aflibercept 2 mg arm did not complete the 1-year follow-up (Fig. 1).

**Table 1:** Baseline characteristics of Faricimab vs. Aflibercept patients.

Characteristic	Faricimab	Aflibercept 2 mg	*P* value
Sample size, n	86	86	
Age, years, mean ± SD	80.0 ± 6.4	79.2 ± 8.2	0.495
Male gender, n (%)	36 (41.9)	33 (38.4)	0.756
Right eye, n (%)	42 (48.8)	42 (48.8)	1.000
MNV type, n (%)			0.884
Type 1	43 (50.0)	45 (52.3)	
Type 2	15 (17.4)	16 (18.6)	
Type 3	28 (32.6)	25 (29.1)	
Baseline IOP, mmHg, mean ± SD	14.6 ± 2.8	14.8 ± 2.7	0.760
Baseline BCVA, logMAR, median (IQR)	0.400 (0.300-0.900)	0.430 (0.250-0.730)	0.177
Baseline imaging findings, n (%)			
IRF present	51 (59.3)	48 (55.8)	0.758
SRF present	72 (83.7)	66 (76.7)	0.338
SHRM present	74 (86.0)	74 (86.0)	1.000
RPE rupture present	3 (3.5)	0 (0.0)	0.244
Dye angiography performed	3 (3.5)	9 (10.5)	0.132
Systemic conditions, n (%)			
Hypertension	35 (40.7)	30 (34.9)	0.529
Diabetes mellitus	18 (20.9)	10 (11.6)	0.148
Hyperlipidaemia	16 (18.6)	18 (20.9)	0.848
Thyroid disease	3 (3.5)	6 (7.0)	0.493
Heart disease	20 (23.3)	16 (18.6)	0.574
Chronic respiratory disease	9 (10.5)	4 (4.7)	0.249
Digestive disease	6 (7.0)	1 (1.2)	0.123
Urinary disease	7 (8.1)	2 (2.3)	0.171
Neurological disease	6 (7.0)	3 (3.5)	0.493
Cancer history	5 (5.8)	6 (7.0)	1.000

Data are presented as mean ± SD and median (IQR) for continuous variables, and n (%) for categorical variables.

*P*-values are from two-sample t-test/Wilcoxon rank-sum test for continuous variables and Pearson’s chi-square/Fisher’s exact test for categorical variables, as appropriate.

*BCVA* best-corrected visual acuity, *IOP* intraocular pressure, *IQR* interquartile range, *IRF* intraretinal fluid, *logMAR* logarithm of the minimum angle of resolution, *MNV* macular neovascularisation, *RPE* retinal pigment epithelium, *SD* standard deviation, *SHRM* subretinal hyperreflective material, *SRF* subretinal fluid.

### Visual acuity outcomes

**Fig. 2:**
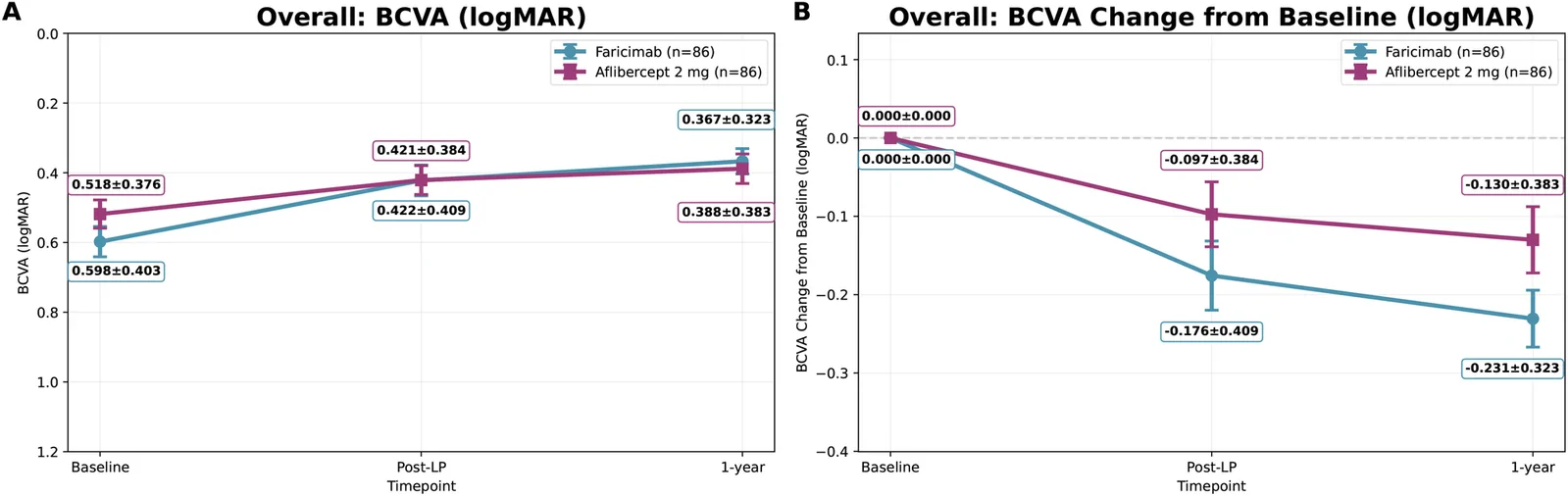
Mean BCVA outcomes in the overall population. **A** Observed mean BCVA (logMAR) over time. **B** Mean change in BCVA (logMAR) from baseline. Results are based on observed data from the intent-to-treat population. Error bars represent the standard error of the mean. The annotation in the upper-right corner indicates the number of eyes available at baseline and post-loading assessments. At the 1-year follow-up, data were available for 79 eyes in the faricimab group and 82 eyes in the aflibercept group. BCVA best-corrected visual acuity, LP loading phase.

Both treatment groups demonstrated improvement in visual acuity from baseline over the follow-up period. After IPTW adjustment, treatment with the faricimab regimen was associated with statistically significantly greater BCVA improvement compared with the aflibercept 2 mg regimen (Fig. 2). In the intent-to-treat analysis, the adjusted mean difference for the BCVA change from baseline to the post-loading phase assessment was -0.075 logMAR (95% confidence interval [CI], -0.130 to -0.020; *P* = 0.008) in favour of faricimab. This greater visual gain was sustained in the cohort with at least one year of follow-up, where the adjusted mean difference in BCVA change from baseline to 1 year was -0.073 (95% CI, -0.128 to -0.018; *P* = 0.011).

### Anatomic outcomes

**Fig. 3:**
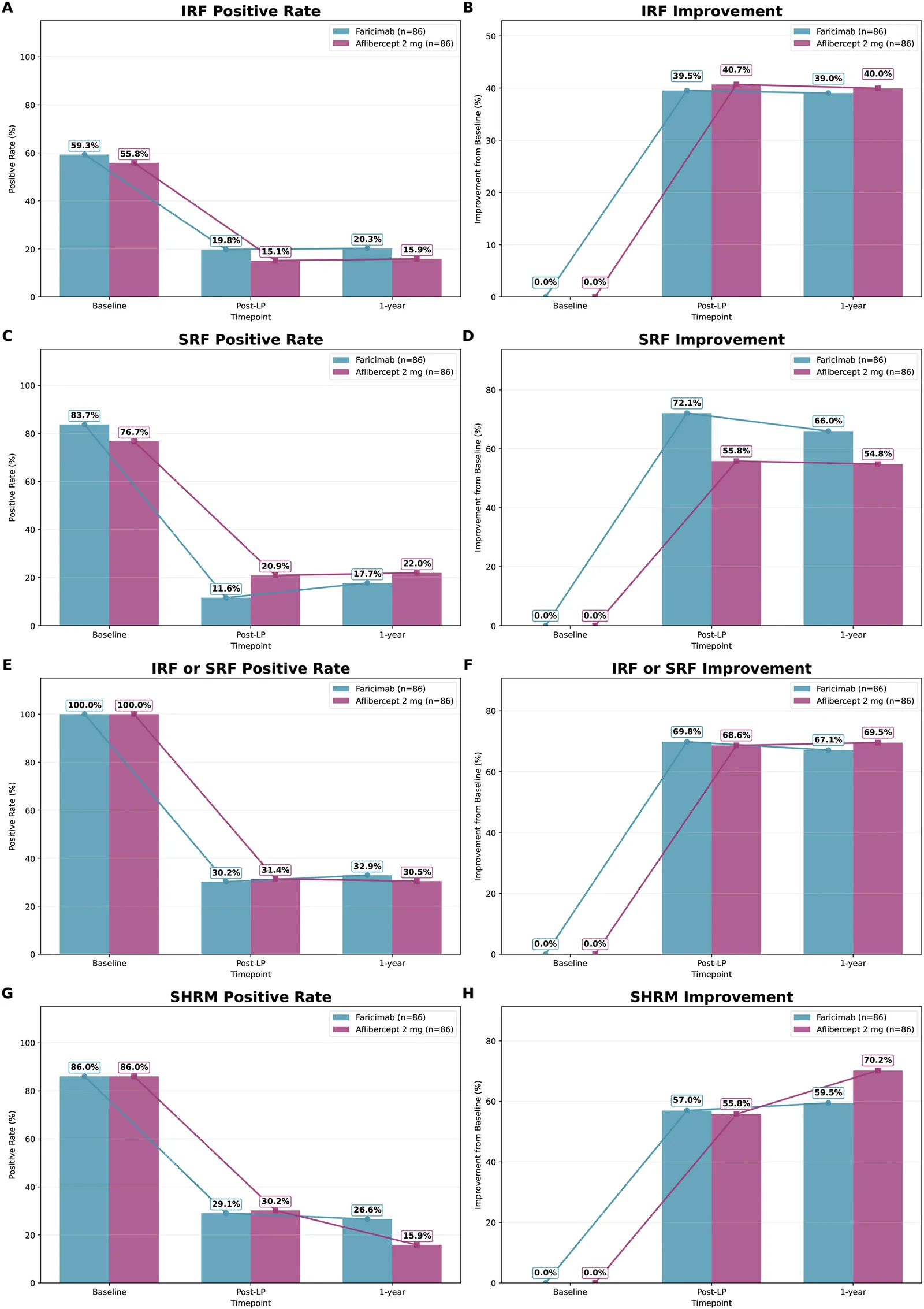
Proportion of patients with retinal morphological features in the overall population. Left panels **A, C, E, G** show the percentage of patients with the presence of IRF, SRF, IRF or SRF, and SHRM at each timepoint. Right panels **B, D, F, H** show the percentage of patients achieving improvement (resolution) of the respective feature from baseline. Results are based on observed data. The annotation in the upper-right corner indicates the number of eyes available at baseline and post-loading assessments. At the 1-year follow-up, data were available for 79 eyes in the faricimab group and 82 eyes in the aflibercept group. IRF intraretinal fluid, SHRM subretinal hyperreflective material, SRF subretinal fluid.

Anatomic outcomes were assessed based on key morphological features on OCT (Fig. 3, Table 2). At baseline, the presence of IRF and SRF was common in both treatment arms. Following treatment initiation, both groups showed a rapid and sustained reduction in the proportion of eyes with these fluid types. When comparing outcomes at the post-loading phase assessment, patients treated with faricimab had a significantly higher likelihood of achieving complete resolution of SRF compared to the aflibercept 2 mg group (odds ratio [OR], 2.446; 95% CI, 1.012 to 5.912; *P* = 0.047). Differences in the proportion of patients achieving resolution of IRF, combined IRF and SRF, or SHRM at two time points were not statistically significant between the two groups.

**Table 2:** IPTW-weighted treatment effects in all patients and in the ≥ 1-year follow-up cohort.

Outcome	Cohort	Faricimab	Aflibercept 2 mg	Effect	*P*_value
BCVA change (Post-BL)	All patients (n = 172)	−0.176 ± 0.182	−0.097 ± 0.194	−0.075 (−0.130, −0.020)	0.008*
IRF improved (Post-BL)	All patients (n = 172)	35 (40.7%)	37 (43.0%)	0.679 (0.272, 1.695)	0.407
SRF improved (Post-BL)	All patients (n = 172)	62 (72.1%)	48 (55.8%)	2.446 (1.012, 5.912)	0.047*
IRF/SRF improved (Post-BL)	All patients (n = 172)	60 (69.8%)	59 (68.6%)	1.152 (0.601, 2.207)	0.671
SHRM improved (Post-BL)	All patients (n = 172)	49 (57.0%)	49 (57.0%)	1.091 (0.551, 2.160)	0.803
BCVA change (1-year)	Follow-up ≥1 y (n = 161)	−0.185 ± 0.178	−0.110 ± 0.187	−0.073 (−0.128, −0.018)	0.011*
IRF improved (1-year)	Follow-up ≥1 y (n = 161)	31 (39.2%)	36 (43.9%)	0.571 (0.217, 1.501)	0.256
SRF improved (1-year)	Follow-up ≥1 y (n = 161)	58 (73.4%)	45 (54.9%)	2.439 (0.998, 5.958)	0.050
IRF/SRF improved (1-year)	Follow-up ≥1 y (n = 161)	54 (68.4%)	56 (68.3%)	1.118 (0.574, 2.179)	0.742
SHRM improved (1-year)	Follow-up ≥1 y (n = 161)	45 (57.0%)	46 (56.1%)	1.162 (0.576, 2.344)	0.674

BCVA change is summarised as mean ± SD; binary imaging outcomes as n (%).

Effects are from IPTW-weighted linear (for BCVA change) and logistic (for binary outcomes) regression models adjusted for baseline BCVA, age, sex, and MNV type. IPTW used stabilised weights with 1st–99th percentile truncation.

*BCVA* best-corrected visual acuity, *IRF* intraretinal fluid, *IPTW* inverse probability of treatment weighting, *MNV* macular neovascularisation, *Post-BL* post-baseline, *SD* standard deviation, *SHRM* subretinal hyperreflective material, *SRF* subretinal fluid.

An asterisk (*) denotes *P* < 0.05.

### Treatment burden and safety

After the initial loading phase, patients in the faricimab arm required a significantly lower number of injections (Supplementary Table 1). The median (interquartile range [IQR]) number of post-loading phase injections was 3 (3–4) for faricimab versus 4 (3–5) for aflibercept 2 mg, with an adjusted mean difference of -0.732 injections (95% CI, -1.130 to -0.333; *P* < 0.001). At 1 year, among the 79 patients treated with faricimab who completed follow-up, 55.7% (44 patients) were maintained on a q16 regimen, 31.6% (25 patients) on q12, and 12.7% (10 patients) on q8. Conversely, among the 82 patients in the aflibercept 2 mg group who completed the 1-year follow-up, 9.8% (8 patients) were on q16, 31.7% (26 patients) on q12, 2.4% (2 patients) on q10, and 56.1% (46 patients) remained on q8.

Furthermore, the rate of switching to an alternative anti-VEGF therapy was significantly lower in the faricimab arm (7.9%) compared to the aflibercept 2 mg arm (25.6%), with an OR of 0.222 (95% CI, 0.081 to 0.604; *P* = 0.003). The total number of injections administered throughout the entire follow-up period was comparable between the two arms (*P* = 0.189). The overall safety profiles were also comparable, with a low incidence of RPE rupture that did not differ significantly between the groups.

### Subgroup analyses

In prespecified subgroup analyses stratified by MNV subtype, no statistically significant differences in BCVA improvement were observed between treatment arms for any subtype (Supplementary Figs. S1–S4). For anatomic outcomes, a significant treatment effect was observed in the MNV Type 3 subgroup at the post-loading phase assessment, where patients receiving faricimab had a significantly higher rate of SRF resolution compared to those on aflibercept 2 mg (60.7% vs. 36.0%; OR 3.502, *P* = 0.011). No other significant differences in anatomic outcomes were found within the MNV Type 1, Type 2, or Type 3 subgroups at any time point.

## Discussion

This retrospective real-world comparative cohort study found that, faricimab was associated with greater BCVA improvement, enhanced anatomic outcomes, fewer post-loading injections, and lower therapy-switching rates than aflibercept 2 mg in treatment-naïve patients with nAMD. These findings suggest that, under the licensed real-world regimens used during the study period, faricimab may provide favourable functional and anatomic outcomes with durable disease control in treatment-naïve nAMD patients.

The findings from our real-world cohort both align with and extend the results of the pivotal TENAYA and LUCERNE trials. The primary endpoint of those trials established that faricimab’s visual acuity gains were non-inferior to those of aflibercept 2 mg at one year [5]. In contrast, our observation of a greater visual outcome suggests a potential advantage in a real-world setting. This difference may arise because faricimab’s potent and sustained anatomical control translates more effectively into functional improvements under the flexible T&E regimens used in routine clinical practice. In strong concordance with trial data, our finding of greater SRF resolution provides a real-world validation of a key post-hoc analysis from the initial head-to-head dosing phase of TENAYA and LUCERNE, which first demonstrated that faricimab achieved a faster and more thorough fluid clearance than aflibercept 2 mg [9].

Notably, we also performed a sub-analysis stratified by MNV type. A significant treatment effect was observed in the Type 3 MNV subgroup at the post-loading phase, with patients receiving faricimab showing a higher rate of SRF resolution compared to those treated with aflibercept 2 mg. Although IRF and SHRM are generally associated with worse visual outcomes [29–32], SRF in Type 3 MNV reflects a later stage of the disease and is linked to poor prognosis [28]. Thus, improved SRF resolution in these patients may indicate a better anatomic outcome.

Importantly, the lower number of post-loading injections observed in our faricimab arm is consistent with the durability demonstrated in the TENAYA and LUCERNE trials; their two-year data showed that over 60% of patients could be maintained on a 16-week dosing interval, with nearly 80% achieving at least a 12-week interval [6]. These extended treatment intervals were shown to maintain visual and anatomical improvements through 48 weeks, supporting the feasibility of less frequent dosing in selected patients [15]. Our real-world findings are consistent with this durability signal, although the lower post-loading injection count should be interpreted cautiously because the faricimab group received one additional loading injection and had an approximately 1-month shorter post-loading observation window; notably, the total number of injections over the entire 1-year follow-up was comparable between groups. Similar findings have been reported in the FARIT trial, a retrospective, observational, multicentre cohort study involving 87 eyes (68 with nAMD and 19 with diabetic macular oedema, DMO) followed for a median of 14 months [33]. Of these, 33 eyes (24 with nAMD and 9 with DMO) were anti-VEGF naïve. After one year, 95.4% of naïve nAMD eyes and 100% of naïve DMO eyes achieved a dosing interval of 12 weeks (Q12W) or longer. The authors reported that faricimab was associated with effective disease control and allowed for extended treatment intervals in both treatment-naïve and previously treated cases of nAMD and DMO, with rapid anatomical response and reduced injection burden.

Our findings contribute to the expanding real-world evidence base for faricimab, which has predominantly examined patients with refractory disease or those switching from alternative anti-VEGF therapies due to suboptimal response or high treatment burden. Multiple studies have demonstrated anatomical improvements and extended treatment intervals when switching treatment-intensive patients to faricimab [18–21]. These switching studies consistently report reductions in central subfield thickness and enhanced fluid resolution, with many patients achieving longer injection intervals ranging from 6.9 to 8.3 weeks compared to their previous regimens [18, 19]. However, visual acuity outcomes in switching populations have generally remained stable rather than showing improvement, likely reflecting the chronic nature of disease in heavily pre-treated eyes [21, 23]. Our study addresses a critical knowledge gap by focusing specifically on treatment-naïve patients, where the potential for visual improvement remains optimal. This distinction is clinically important because treatment-naïve eyes represent the ideal population for establishing first-line therapy superiority. Our results align with a recent indirect treatment comparison that similarly suggested potential benefits of faricimab over standard-of-care anti-VEGF therapy in treatment-naïve Italian patients, demonstrating greater visual gains and anatomical improvements in a real-world setting [34].

A biologically plausible explanation for the observed outcomes is faricimab’s dual inhibition of VEGF-A and angiopoietin-2 pathways. The angiopoietin-2/Tie2 pathway plays a critical role in vascular stability, with angiopoietin-2 acting as a context-dependent antagonist that promotes vascular destabilisation under pathological conditions [35]. By simultaneously blocking angiopoietin-2, faricimab enhances endothelial barrier function and reduces vascular permeability beyond what VEGF-A inhibition alone can achieve [36]. The enhanced anatomical control observed in our study, particularly the greater subretinal fluid resolution, likely reflects the complementary effects of dual pathway inhibition on choroidal neovascular stability and permeability [37]. Angiopoietin-2 inhibition works synergistically with VEGF-A blockade to restore endothelial cell junction integrity and reduce inflammatory vessel leakage [38]. However, the observed differences may also be explained by other factors, including higher molar exposure, pharmacokinetic or pharmacodynamic differences, binding-property differences, and greater early treatment exposure related to the additional faricimab loading injection. Nevertheless, better anatomical control may subsequently facilitate extended treatment intervals, as more stable vascular architecture with reduced leakage propensity may support longer periods between injections without compromising disease control.

Our study benefits from several methodological strengths while acknowledging important limitations. The real-world design captures clinical practice heterogeneity beyond controlled trial environments, and the focus on treatment-naïve patients addresses a critical evidence gap, as most real-world faricimab studies examine switching scenarios. IPTW improved balance in measured baseline characteristics; however, the retrospective, non-randomised, single-centre design remains vulnerable to residual confounding, particularly because treatment allocation partly reflected drug availability over calendar time. In addition, the comparison reflected the licensed regimens used during the study period, with four initial monthly faricimab injections versus three initial monthly aflibercept 2 mg injections. This difference in early treatment exposure may have influenced post-loading outcomes and subsequent treatment burden, and the retrospective design cannot disentangle the relative contributions of loading intensity, molar drug exposure, pharmacologic properties, and dual Ang-2/VEGF-A inhibition. Finally, this study did not include the newer aflibercept 8 mg formulation; therefore, the findings should not be extrapolated to comparisons with high-dose aflibercept.

These findings support faricimab as a clinically relevant first-line option for treatment-naïve neovascular AMD patients under licensed real-world treatment regimens. The observed functional, anatomical, and treatment-burden outcomes are encouraging, but should be interpreted in light of the retrospective design, differences in loading regimens and drug exposure, and the absence of a comparison with aflibercept 8 mg. Future research should prioritise randomised or appropriately controlled studies with harmonised loading exposure, longer-term follow-up, multicentre validation, cost-effectiveness analyses incorporating treatment burden, and biomarker investigation for personalised treatment approaches across MNV subtypes.

Supplemental material is available at Eye’s website.

## Summary

### What was known before

- Faricimab represents a novel therapeutic strategy as the first bispecific antibody designed for intraocular use, specifically targeting and inhibiting two key pathogenic pathways: angiopoietin-2 (Ang-2) and vascular endothelial growth factor A (VEGF-A).
- A well-known gap exists between the controlled conditions of randomised clinical trials (RCTs) and the complexities encountered in routine clinical practice.
- There remains a significant knowledge gap regarding the real-world comparative effectiveness of faricimab versus aflibercept 2 mg as first-line treatments for treatment-naïve neovascular AMD.

### What this study adds

- This real-world comparative cohort study shows that faricimab provides superior therapeutic outcomes compared with aflibercept 2 mg when used as first-line therapy in treatment-naïve patients with nAMD.
- Our findings indicate that the durability observed in clinical trials is also reflected in real-world clinical practice.

## Supplementary information

Supplemental Table 1

Supplemental Figure 1

Supplemental Figure 2

Supplemental Figure 3

Supplemental Figure 4

## Data Availability

Data that support the findings of this study are available upon reasonable request to the corresponding author.
