# Supplementary material for: Real-world comparative effectiveness of faricimab versus aflibercept 2 mg in treatment-naïve exudative neovascular AMD patients treated with a treat-and-extend regimen
Source: Eye (Lond). 2026 Aug 4;40(15):2367–74. doi: 10.1038/s41433-026-04710-y (PMC13675230; doi:10.1038/s41433-026-04710-y)
Supplement: Supplementary file 1 [file 41433_2026_4710_MOESM1_ESM.pdf]

Supplementary Table 1. Treatment burden and safety in all patients and in the ≥1-year follow-up cohort

Outcome	Cohort	Faricimab	Aflibercept 2 mg	Effect	P_value
Number of injection after LP	Follow-up ≥1y (n=161)	3.0 (3.0-4.0)	4.0 (3.0-5.0)	-0.732 (-1.130, -0.333)	<0.001*
Total injections	Follow-up ≥1y (n=161)	7.0 (7.0-8.0)	7.0 (6.0-8.0)	0.268 (-0.130, 0.667)	0.189
Drug Switch after LP	Follow-up ≥1y (n=161)	6 (7.9%)	21 (25.6%)	0.222 (0.081, 0.604)	0.003*
Endophthalmitis after post-LP	All patients (n=172)	0 (0.0%)	0 (0.0%)	N/A	N/A
Endophthalmitis within 1 year	Follow-up ≥1y (n=161)	0 (0.0%)	0 (0.0%)	N/A	N/A
RPE rupture developed after post-LP	All patients (n=172)	1 (1.2%)	0 (0.0%)	2.457 (0.222, 27.167)	0.463
RPE rupture developed within 1 year	Follow-up ≥1y (n=161)	1 (1.3%)	0 (0.0%)	2.628 (0.237, 29.131)	0.431

Number of injections after last procedure and total injections are summarized as median (IQR); binary treatment burden and safety outcomes (drug switch, endophthalmitis, RPE rupture) as n (%).

Effects are from IPTW-weighted linear regression (for injection counts) and logistic regression (for drug switch, endophthalmitis and RPE rupture) adjusted for baseline BCVA, age, sex, and MNV type. IPTW used stabilized weights with 1st–99th percentile truncation.

BCVA = best-corrected visual acuity; IQR = interquartile range; IPTW = inverse probability of treatment weighting; LP = loading phase; MNV = macular neovascularization; RPE = retinal pigment epithelium.

An asterisk (*) denotes P < 0.05.
