# Supplementary material for: Real-world comparative effectiveness of faricimab versus aflibercept 2 mg in treatment-naïve exudative neovascular AMD patients treated with a treat-and-extend regimen
Source: Eye (Lond). 2026 Aug 4;40(15):2367–74. doi: 10.1038/s41433-026-04710-y (PMC13675230; doi:10.1038/s41433-026-04710-y)
Supplement: Supplementary file 2 [file 41433_2026_4710_MOESM2_ESM.pdf]

Supplementary Figure 1. Mean BCVA outcomes stratified by MNV subtype.

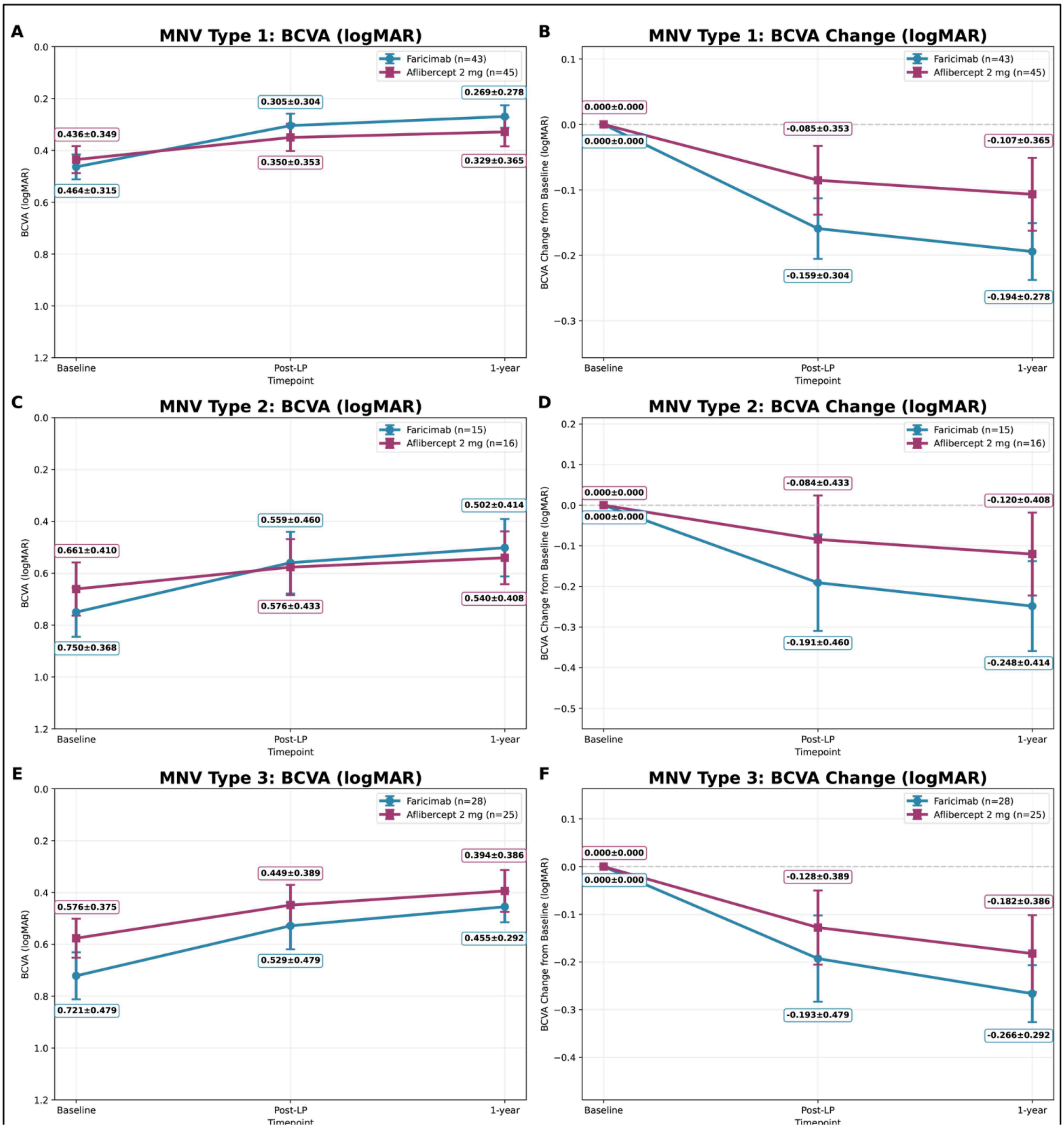

(A, C, E) Observed mean BCVA (logMAR) over time for MNV Type 1, Type 2, and Type 3, respectively. (B, D, F) Mean change in BCVA (logMAR) from baseline for MNV Type 1, Type 2, and Type 3, respectively. Results are based on observed data from the intent-to-treat population. Error bars represent the standard error of the mean. BCVA = best-corrected visual acuity; LP = loading phase; MNV = macular neovascularization. The annotation in the upper-right corner of each panel indicates the number of eyes available for the corresponding MNV subtype at baseline and post-loading assessments. Sample sizes by MNV subtype were as follows: Type 1, baseline/post-loading faricimab n=43 and aflibercept n=45, and 1-year faricimab n=41 and aflibercept n=43; Type 2, baseline/post-loading faricimab n=15 and aflibercept n=16, and 1-year faricimab n=14 and aflibercept n=16; Type 3, baseline/post-loading faricimab n=28 and aflibercept n=25, and 1-year faricimab n=24 and aflibercept n=23.
