# Supplementary material for: Real-world comparative effectiveness of faricimab versus aflibercept 2 mg in treatment-naïve exudative neovascular AMD patients treated with a treat-and-extend regimen
Source: Eye (Lond). 2026 Aug 4;40(15):2367–74. doi: 10.1038/s41433-026-04710-y (PMC13675230; doi:10.1038/s41433-026-04710-y)
Supplement: Supplementary file 5 [file 41433_2026_4710_MOESM5_ESM.pdf]

**Supplementary Figure 4. Proportion of patients with retinal morphological features in MNV Type 3.**

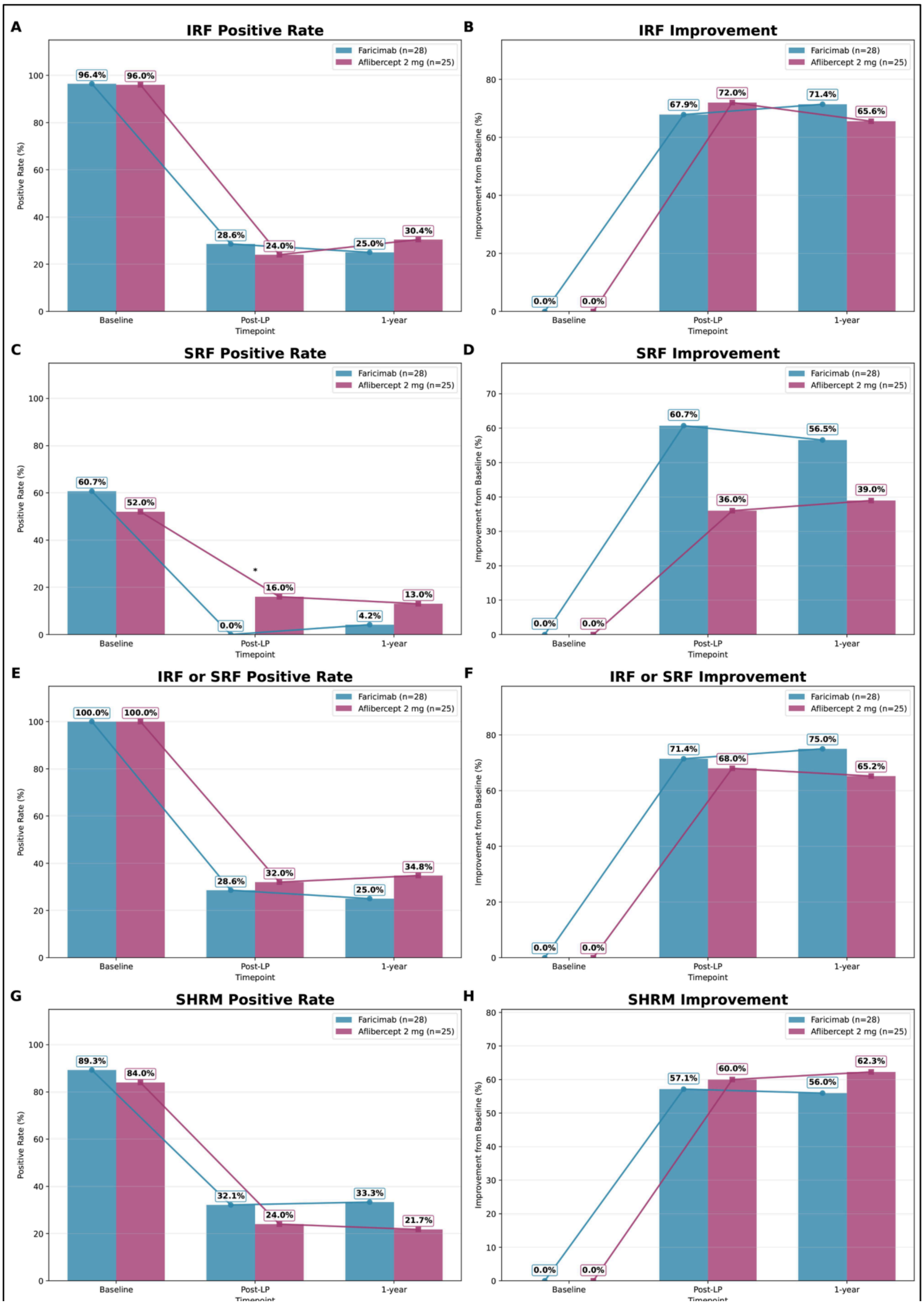


Left panels (A, C, E, G) show the percentage of patients with the presence of IRF, SRF, IRF or SRF, and SHRM at each timepoint. Right panels (B, D, F, H) show the percentage of patients achieving improvement (resolution) of the respective feature from baseline. Results are based on observed data from MNV Type 3 patients. IRF = intraretinal fluid; MNV = macular neovascularization; SHRM = subretinal hyperreflective material; SRF = subretinal fluid. The annotation in the upper-right corner of each panel indicates the number of eyes available for MNV type 3 at baseline and post-loading assessments. For MNV type 3, data were available for 28 faricimab-treated eyes and 25 aflibercept-treated eyes at baseline and post-loading assessments, and for 24 faricimab-treated eyes and 23 aflibercept-treated eyes at the 1-year follow-up.
